# Characterization of the histone H2A.Z-1 and H2A.Z-2 isoforms in vertebrates

**DOI:** 10.1186/1741-7007-7-86

**Published:** 2009-12-14

**Authors:** Deanna Dryhurst, Toyotaka Ishibashi, Kristie L Rose, José M Eirín-López, Darin McDonald, Begonia Silva-Moreno, Nik Veldhoen, Caren C Helbing, Michael J Hendzel, Jeffrey Shabanowitz, Donald F Hunt, Juan Ausió

**Affiliations:** 1 Department of Biochemistry and Microbiology and The Center for Biomedical Research, University of Victoria, Petch Building, Victoria, BC, V8W 3P6, Canada; 2 California Institute for Quantitative Biosciences, University of California, Berkeley, 642 Stanley Hall, Berkeley, CA, 94720-3220, USA; 3 Department of Chemistry, University of Virginia, Charlottesville, VA, 22904, USA; 4 The Scripps Research Institute, 130 Scripps Way #1B2, Jupiter, FL, 33458, USA; 5 Departamento de Biología Celular y Molecular, Universidade da Coruña, E15071 A Coruña, Spain; 6 Department of Oncology, University of Alberta and Cross Cancer Institute, Edmonton, Alberta, T6G 1Z2, Canada; 7 Department of Pathology, University of Virginia, Charlottesville, VA, 22904, USA

## Abstract

**Background:**

Within chromatin, the histone variant H2A.Z plays a role in many diverse nuclear processes including transcription, preventing the spread of heterochromatin and epigenetic transcriptional memory. The molecular mechanisms of how H2A.Z mediates its effects are not entirely understood. However, it is now known that H2A.Z has two protein isoforms in vertebrates, H2A.Z-1 and H2A.Z-2, which are encoded by separate genes and differ by 3 amino acid residues.

**Results:**

We report that H2A.Z-1 and H2A.Z-2 are expressed across a wide range of human tissues, they are both acetylated at lysine residues within the N-terminal region and they exhibit similar, but nonidentical, distributions within chromatin. Our results suggest that H2A.Z-2 preferentially associates with H3 trimethylated at lysine 4 compared to H2A.Z-1. The phylogenetic analysis of the promoter regions of H2A.Z-1 and H2A.Z-2 indicate that they have evolved separately during vertebrate evolution.

**Conclusions:**

Our biochemical, gene expression, and phylogenetic data suggest that the H2A.Z-1 and H2A.Z-2 variants function similarly yet they may have acquired a degree of functional independence.

## Background

In the eukaryotic cell, the template for all DNA metabolic activities including DNA repair, replication, recombination and transcription, is chromatin. Chromatin is a nucleoprotein complex in which approximately 147 base pairs of DNA are wrapped around a histone core consisting of two each of the core histones H2A, H2B, H3 and H4, resulting in a repetitive structure called the nucleosome. Linker histones of the H1 family bind to the linker DNA regions connecting adjacent nucleosomes in the chromatin fiber. Histones represent the major protein component of chromatin and most of the synthesis of the canonical forms takes place during S phase of the cell cycle to allow for efficient packaging of the newly replicated DNA. In contrast, a subset of histone variants is synthesized throughout the cell cycle and can replace canonical histones to specify chromatin domains for specific functions [1,2]. Histone H2A.Z is one such replacement histone variant and while it is very widely studied, its structural and functional roles have not only proven to be many and diverse but also controversial [2,3].

From a functional perspective, histone H2A.Z has been found to be present in heterochromatin, where it participates in the formation of pericentric and centric chromatin, [4,5] and in euchromatin [6,7], where it is usually found associated with promoters of active genes [8]. Recent work has revealed that promoters are marked by complexes that contain H2A.Z in conjunction with the H3.3 variant indicating that replication-independent histone variant replacement plays a role in generating an appropriate chromatin landscape at these loci [9]. The concept that H2A.Z serves to poise promoters for transcriptional activation but is displaced from chromatin once transcription is initiated has been widely documented [10-13].

Recently, another very interesting function has been ascribed to H2A.Z, which is that it aids in localizing genes to the nuclear periphery and in doing so marks them for more rapid reactivation even after several cell divisions [14]. Many of these functions of H2A.Z, although seemingly very different, may not be entirely unrelated in a broad sense if H2A.Z is considered a mark that is required to specify the nuclear location of a given region of the genome perhaps with respect to the nuclear periphery, or the nuclear matrix in general.

At the structural level, H2A.Z has been shown to enhance the stability of the nucleosome [15,16] despite the fact that the H2A.Z-H2B dimer exhibits a reduced stability compared to H2A-H2B dimers [16,17]. H2A.Z has also been shown to alter nucleosome mobility [18]. When present in nucleosome arrays, H2A.Z increases the intramolecular interactions and promotes HP1α-mediated folding of the fiber [19,20]. Furthermore, several well-positioned H2A.Z-containing nucleosomes flank sites occupied by the insulator binding protein CTCF which prevents the spread of heterochromatin [21].

How H2A.Z is able to participate in these many cellular events could be based on whether it is placed within isolated nucleosomes or within contiguous stretches of chromatin, as is the case at many polycomb group genes in embryonic stem cells [22]. Alternatively, post translational modifications shown to exist on H2A.Z, including acetylation, ubiquitination and SUMOylation, may direct certain populations of H2A.Z for a specific function.

Recently, we identified the presence of two H2A.Z protein isoforms in chicken that differ by 3 amino acids [23]. Our group has also provided evidence of distinct phylogenetic patterns for the H2A.Z-1 and H2A.Z-2 variants during vertebrate evolution [24]. Importantly, H2A.Z is the only histone variant that has been shown to be indispensable for survival in Drosophila [25] and mice [26]. However, in this latter study, only H2A.Z-1 was knocked out. This indicates that H2A.Z-2 is incapable of compensating for the loss of H2A.Z-1 in mice. Whether this is because of lower amounts of total H2A.Z or because of differences in the nuclear localization, post translational modification, biochemical interactions or temporal expression of the H2A.Z-1 and H2A.Z-2 genes is unknown. In the present work, we show that both isoforms are expressed across a wide range of human tissues and that they display a similar nuclear distribution and levels of N-terminal acetylation. Furthermore, we show that the distribution of H2A.Z-1 and H2A.Z-2 within chromatin differs, as does their association with histone H3 trimethylated at lysine 4. Despite the high degree of amino acid sequence similarity between these H2A.Z isoforms, they display very divergent promoter sequences that could result in temporal and tissue-specific differences in gene expression.

## Results

### The N-terminal tails of H2A.Z-1 and H2A.Z-2 are acetylated *in vivo *in chicken cells

**Figure 1:**
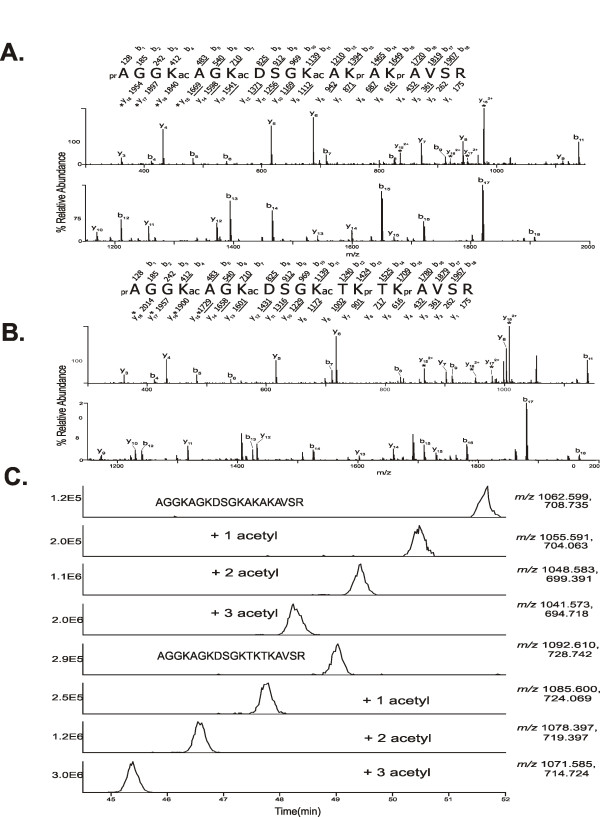
**(A)** Mass spectrometry (MS/MS) spectrum of the N-terminal H2A.Z-2 peptide, AGGKAGKDSGKAKAKAVSR. The peptide is modified with three acetyl groups on lysines 4, 7, and 11. **(B)** MS/MS spectrum of the triply-acetylated N-terminal peptide, AGGKAGKDSGKTKTKAVSR, of H2A.Z-1. The acetyl groups were again identified on lysines 4, 7, and 11. The precursor ions selected for dissociation were the [M+2H]^+2 ^ions, and are m/*z *1041.1 **(A)** and m/*z *1071.1 **(B)**, respectively. The amino acid sequences are shown above the spectra, and the masses above and below the sequences correspond to the theoretical b- and y-type product ions. The masses provided are the monoisotopic, nominal masses of the product ions. The observed, singly-protonated b- and y-type ions are underlined and are assigned to their corresponding m/*z *peaks in the spectra. The observed, doubly-protonated ions are denoted with asterisks. The acetylated lysines (K4, K7, and K11) are indicated with 'ac'. The unacetylated amino groups were derivatized with propionic anhydride and are denoted with 'pr'. **(C)** Selected ion chromatograms (SICs) for the N-terminal peptides of H2A.Z-2 and H2A.Z-1. The theoretical m/*z *values of the [M+2H]^+2 ^and [M+3H]^+3 ^ions for each peptide were used to generate the SICs and these values are adjacent to each chromatogram. Note that the retention time decreases with increasing number of acetyl groups due to the loss of propionylated lysine. The ion count intensities for each SIC are located on the *y*-axis and are provided on the left adjacent to each chromatogram.

We employed a mass spectrometric approach in order to determine if the N-terminal tail of H2A.Z-1 is acetylated *in vivo *in a similar way to the recently described acetylation of the same region in H2A.Z-2 [27]. Total H2A.Z protein was purified from chicken erythrocytes and sodium butyrate-treated MSB cells by a combination of gel filtration chromatography and RP-HPLC. Purified H2A.Z was then derivatized with propionic anhydride to limit trypsin digestion to arginine residues as previously described [27]. The H2A.Z peptides were next analysed by LC-coupled tandem mass spectrometry which enabled sequence determination of the first 19 residues of both H2A.Z-1 (AGGKAGKDSGKTKTKAVSR) and H2A.Z-2 (AGGKAGKDSGKAKAKAVSR). This technique also afforded the identification of multiple acetylation sites on both of the N-terminal peptides from the sodium butyrate-treated sample. Figure 1A and 1B illustrate the mass spectrometry (MS/MS) spectra of the most abundant form of the N-terminal peptide identified in the sodium butyrate-treated sample which was a triply-acetylated form with acetylation present on K4, K7 and K11. In fact, four different forms of the H2A.Z-1 N-terminal peptide were detected, similar to those previously described for H2A.Z-2 [27]. Selected ion chromatograms, shown in Figure 1C, illustrate the presence and abundance of the unmodified, singly-acetylated, doubly-acetylated and triply-acetylated peptides from both H2A.Z isoforms. Albeit at lower relative abundance, doubly-acetylated species were also enriched in this sample and include forms concurrently acetylated at K4 + K7, K7 + K11 and K4 + K11 for both isoforms. Singly-acetylated species were also detected but were present at significantly lower abundance and, as a consequence, the acetylation sites were not able to be determined for these forms. However, it is likely that they are very similar to those previously determined for H2A.Z-2, since all other modified forms are also very similar for both isoforms.

### H2A.Z-1 and H2A.Z-2 are mainly distributed in euchromatin in mouse fibroblasts and in HEK 293 cells

**Figure 2:**
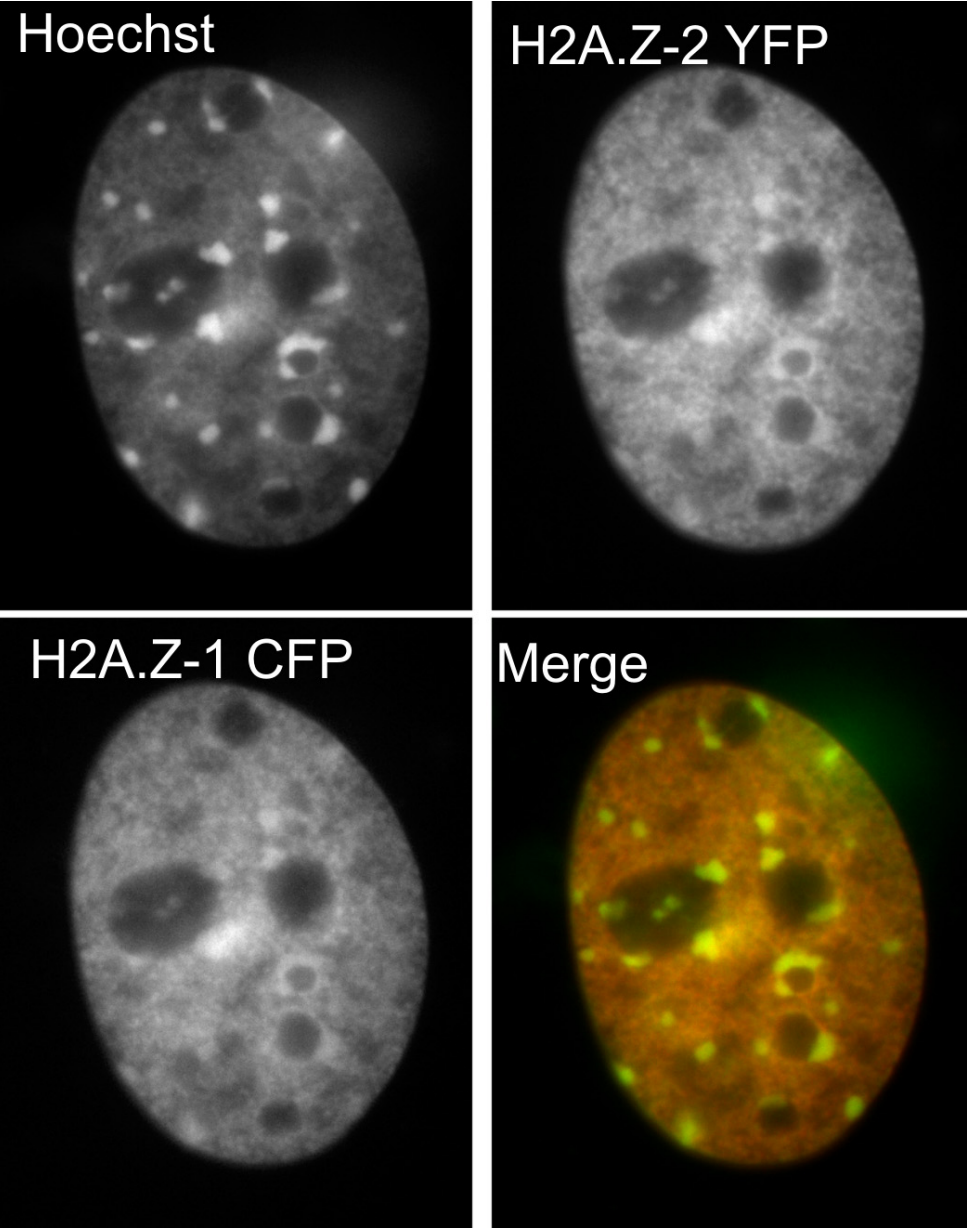
**Fluorescence microscopy of H2A.Z-1 and H2A.Z-2 variants in mouse embryonic fibroblasts**. A mouse embryonic fibroblast nucleus is shown following transfection with H2A.Z-2-YFP and H2A.Z-1-cyan fluorescent protein (CFP) and imaged live. The top left panel shows cells stained with DNA binding dye Hoechst 33342. The top right panel shows the distribution of H2A.Z-2-yellow fluorescent protein (YFP). The bottom left panel shows the distribution of H2A.Z-1-CFP while the composite image shows the Hoechst staining (green) relative to the distribution of H2A.Z-2-YFP. Sites that are enriched in H2A.Z-2 are orange and are predominantly euchromatin. The chromocenters appear yellow, indicating the presence of both DNA and H2A.Z-2-YFP.

In order to determine the pattern of H2A.Z-1 and H2A.Z-2 distribution within chromatin, asynchronous mouse embryonic fibroblasts were transfected with YFP-H2A.Z-2 and CFP-H2A.Z-1 and imaged live (Figure 2). Figure 2 indicates that YFP-H2A.Z-2 (top right panel) and CFP-H2A.Z-1 (bottom left panel) have a near identical distribution throughout chromatin. The composite image showing Hoechst DNA staining (green) and YFP-H2A.Z-2 (red) indicates H2A.Z-2 is preferentially located within regions of euchromatin (orange). However, since both variants localize to the same regions, it stands that H2A.Z-1 would also be mainly present in euchromatic regions. Both variants, however, are also present within the DNA-dense chromocenters, as shown by their yellow staining in the composite image. To ensure that the transfected proteins were able to be incorporated into nucleosomes, chromatin from these cells was isolated, digested with microccocal nuclease and separated on sucrose gradients in order to obtain mononucleosomes [28]. Western blot analysis of these mononucleosomes with an anti-GFP antibody confirmed the incorporation of the transfected H2A.Z variants (data not shown).

**Figure 3:**
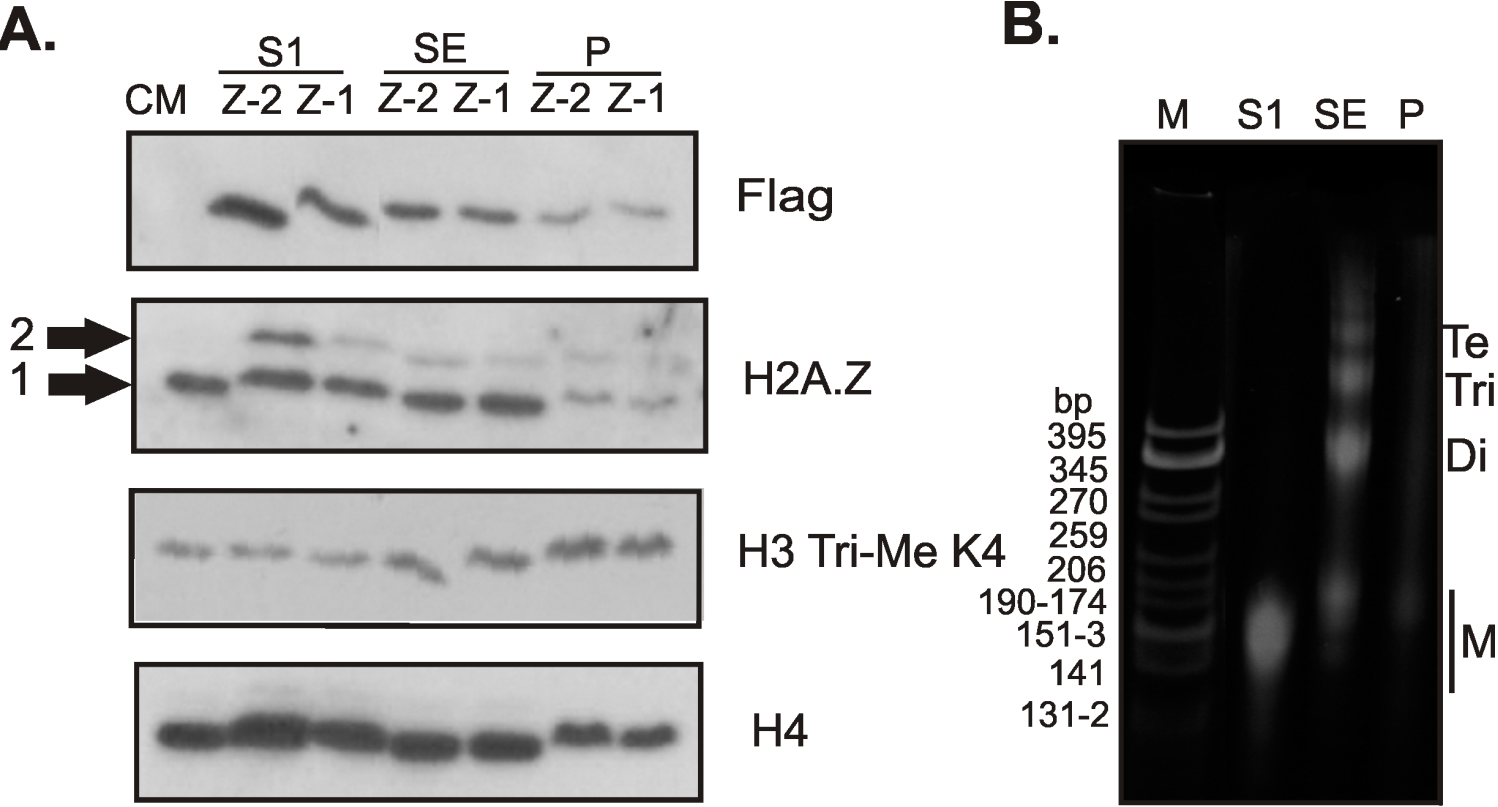
**Distribution of H2A.Z-2 and H2A.Z-1 within chromatin fractions**. (A) S1, SE and P chromatin fractions were generated from HEK 293 cells stably expressing Flag epitope tagged H2A.Z-2 and H2A.Z-1, resolved by sodium dodecyl sulphate-PAGE and analysed by Western blot. The antibodies used for Western blotting are indicated on the right. The anti-H2A.Z antibody recognizes both isoforms in their endogenous (arrow 1) and Flag-tagged (arrow 2) forms. Probing with the anti-H3 Tri Me K4 antibody indicates that this modification is more enriched within the P fraction and therefore does not partition equally among the fractions. Total histone H4 was used as a loading control and CM indicates chicken erythrocyte histone marker. The trends seen were consistent across multiple experimental replicates. (B) 4% native acrylamide gel of purified DNA from the S1, SE and P chromatin fractions used in A. The S1 fraction contains mononucleosomes (M) with approximate DNA length 150 bp, while the SE and P fractions contain chromatin composed of mononucleosomes, dinucleosomes (Di), trinucleosomes (Tri), tetranucleosomes (Te) and longer chromatin. M is CFO-1 cut pBR322 DNA marker.

In order to analyse the distribution of H2A.Z variants within chromatin biochemically, C-terminal Flag epitope tagged H2A.Z-1 and H2A.Z-2 were stably transfected into HEK 293 cells, nuclei were isolated and digested with microccocal nuclease and the chromatin was separated into S1, SE and Pellet fractions. The stably transfected clones that were selected had nearly identical expression levels of the respective Flag-tagged H2A.Z that represented less than 30% of the total endogenous H2A.Z (Figure 3A and data not shown). The highly nuclease-accessible and low salt soluble S1 fraction contains mainly mononucleosomes having a DNA length of approximately 146 bp that generally represents active chromatin, while the more nuclease-resistant SE fraction contains mainly repressed chromatin with DNA of varying lengths (Figure 3B) [29]. The insoluble Pellet fraction most likely represents a mixture of transcriptionally active and repressed chromatin that is insoluble due to its association with large protein complexes such as the RNA Pol II and chromatin remodelling complexes or components of the nuclear matrix (Figure 3B) [29]. This fraction contains far fewer histones compared to other high molecular weight proteins. Western blot analysis of the histones extracted from these fractions, using an antibody against H2A.Z that does not discriminate between the variants, indicates that total endogenous H2A.Z (bottom band, arrow 1) is present in all three chromatin fractions but is more abundant in the S1 and SE compared to the P (Figure 3A). This antibody is also able to detect the Flag-tagged H2A.Z proteins (top band, arrow 2) and indicates that, although these forms fractionate similarly to the endogenous forms, the H2A.Z-2-Flag protein is present in higher amounts in the S1 fraction. This pattern can also be seen when the blot is probed with an anti-Flag antibody (Figure 3A). These blots were further probed with an antibody against total H4 as a loading control and against H3 trimethylated at lysine 4 (H3 Tri-Me K4). Staining with the latter antibody indicates that this modification does not partition equally among the fractions, but is proportionally more abundant within the pellet fraction. Since H3 Tri-Me K4 is a marker of promoter regions of active genes, this result is in agreement with the notion that the Pellet fraction contains genomic regions that are actively being transcribed.

### H2A.Z-1 and H2A.Z-2 associate with different forms of post-translationally modified H3 and H4 within the nucleosome

**Figure 4:**
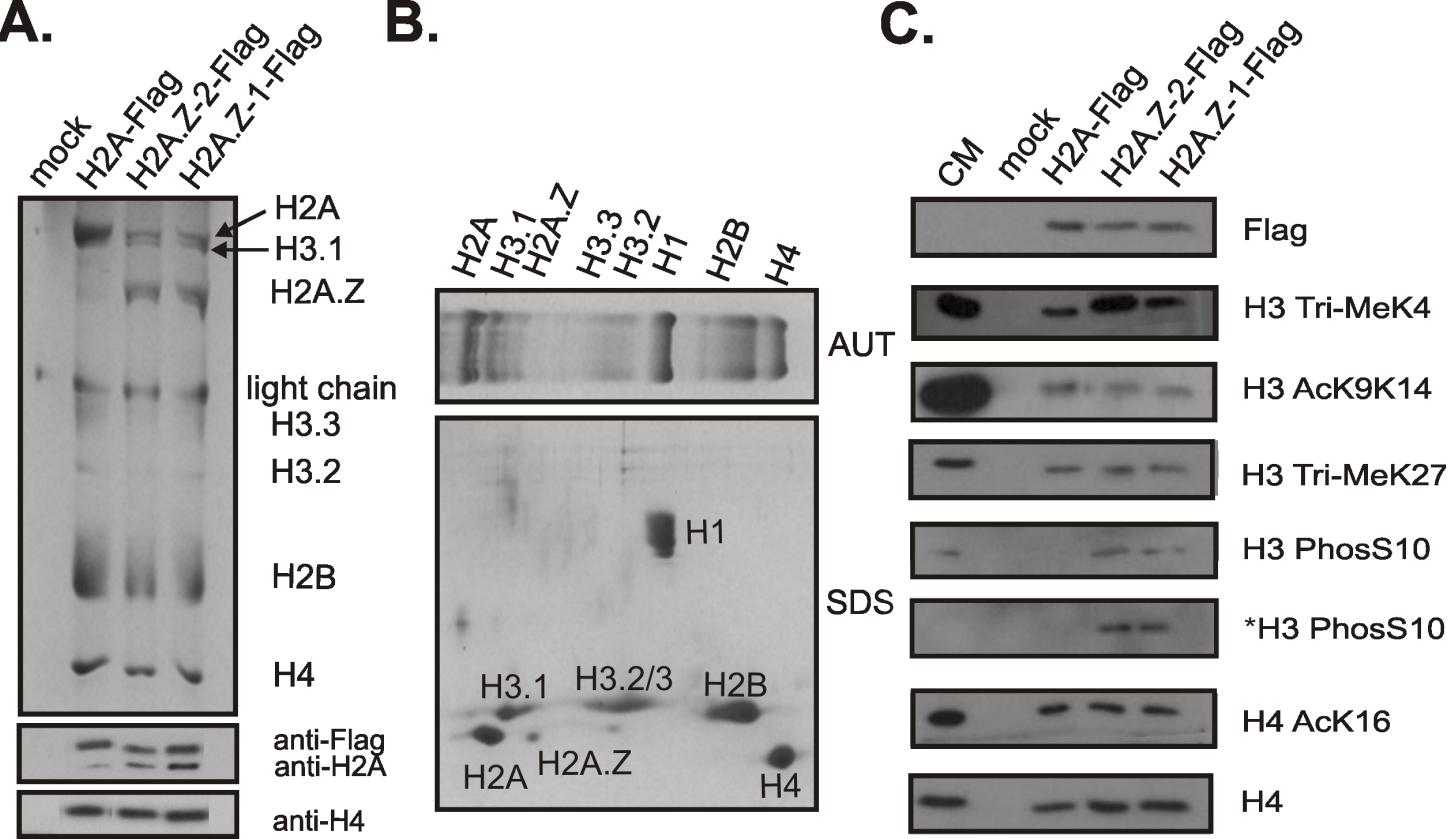
**Immunoprecipitation of H2A.Z-2- and H2A.Z-1-containing mononucleosomes**. (A) Top panel: acid-urea-triton (AUT)-PAGE of the histones from HeLa cell mononucleosomes immunoprecipitated with anti-Flag agarose beads. Light chain refers to the immunoglobulin light chain from the anti-Flag agarose beads. Bottom panels: Western blots of anti-Flag, anti-H2A and anti-H4 (loading control) for the histones from immunoprecipitated mononucleosomes separated by sodium dodecyl sulphate (SDS)-PAGE. B.) Two-dimensinal PAGE analysis of HeLa cell histones with the first dimension AUT shown on top and second dimension SDS gel shown below. (C) Western blots of the histones from anti-Flag agarose bead immunoprecipitated mononucleosomes electrophoresed on 15% SDS gels. Samples were normalized with respect to Flag and total H4 levels. Antibodies used to probe the Western blots are indicated on the right. The asterisk indicates the cells were arrested in mitosis by nocodazole treatment prior to immunoprecipitation. The trends in the association of the Flag-tagged proteins with post-translationally modified forms of H3 and H4 were consistent across multiple replicates of the experiment of which a representative example is shown.

We next sought to determine if the H2A.Z variants differentially associate with several post-translationally modified forms of other histones within the nucleosome. Following the protocol of Sarcinella and colleagues [30], C-terminal Flag-tagged versions of H2A.Z-1, H2A.Z-2 and H2A as a control were transiently expressed in HeLa cells and the chromatin was digested to mononucleosomes using micrococal nuclease. Each preparation of mononucleosomes was analysed on native acrylamide gels in order to ensure complete digestion of the chromatin (data not shown). The nucleosomes containing the Flag-tagged proteins were immunoprecipitated using anti-Flag agarose beads and the specificity of the immunoprecipitations was monitored by AUT-PAGE (polyacrylamide gel electrophoresis; Figure 4A). Figure 4A shows that the immunoprecipitations are specific for H2A-Flag, H2A.Z-2-Flag or H2A.Z-1-Flag containing nucleosomes. The identity of these histones was further confirmed by two-dimensional PAGE with an acid-urea-triton (AUT) gel in the first dimension followed by a sodium dodecyl sulphate (SDS) gel in the second dimension (Figure 4B). Figure 4A also indicates that a proportion of both the H2A.Z-1-Flag and H2A.Z-2-Flag nucleosomes only contain one copy of the tagged protein in the histone octamer, as an H2A band with equal staining intensity is also present in the gel. In order to confirm this result, SDS-PAGE followed by a Western blot using an anti-H2A antibody was performed on the histones from the immunoprecipitated mononucleosomes and is shown in the bottom panels of Figure 4A. Bands corresponding to H2A are present in all three normalized mononucleosome preparations but with a higher intensity in the H2A.Z-2-Flag and H2A.Z-1-Flag nucleosomes (Figure 4A, bottom panels). The presence of these heterotypic H2A.Z nucleosomes has been previously documented in HeLa cells by mass spectrometry [31]. The histones of the immunoprecipitated nucleosomes that were normalized with respect to total H4 were resolved by SDS-PAGE and transferred to membranes which were probed with antibodies specific for several post-translationally modified forms of histones (Figure 4C). Both forms of H2A.Z nucleosomes are enriched in H3 trimethylated at lysine 4 compared to H2A nucleosomes as shown by other groups (Figure 4C) [30,31]. However, this enrichment is greater in the case of the H2A.Z-2-Flag nucleosomes than in the H2A.Z-1-Flag nucleosomes. The promoters of most protein coding genes contain nucleosomes that have the H3 Tri-Me K4 mark along with H3 acetylated at K9 and K14 [32,33]. When the immunoprecipitated nucleosomes were probed with an antibody against this latter H3 modification, roughly equal amounts can be seen in the H2A-Flag, H2A.Z-2-Flag and H2A.Z-1-Flag nucleosomes (Figure 4C). H3 trimethylated at lysine 27 is a marker of inactive promoters and mediates transcriptional silencing [34]. The levels of this modification are relatively equal among all the Flag immunoprecipitated nucleosomes (Figure 4C). Similarly, the levels of H4 acetylated at lysine 16 are equal among nucleosomes. Interestingly, H2A.Z-1-Flag and H2A.Z-2-Flag nucleosomes are enriched in H3 phosphorylated at serine 10 compared to H2A-Flag nucleosomes. This pattern was also seen when the transfected cells were arrested in mitosis by nocodazole treatment before generation of mononucleosomes and immunoprecipitation. It is possible that the majority of the H3PhosS10 staining in the asynchronous cells could be due to the proportion of mitotic cells within that population. This is probably the case, since the staining intensity is increased in mitotic H2A.Z-1 and H2A.Z-2 nucleosomes.

### H2A.Z-1 and H2A.Z-2 are differentially expressed among tissues

**Figure 5:**
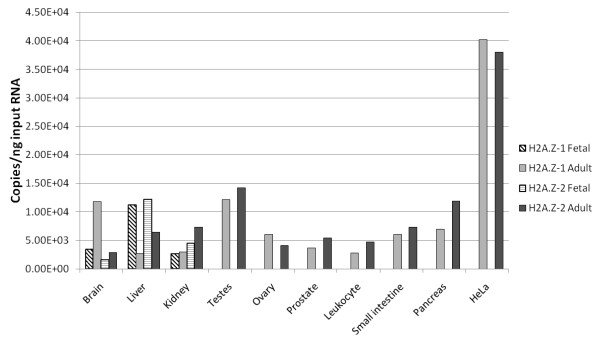
**Quantitative polymerase chain reaction analysis of H2A.Z-1 and H2A.Z-2 mRNA transcript levels in adult and fetal human tissues**. The abundances of H2A.Z-1 and H2A.Z-2 transcript levels were determined relative to a standard curve of known DNA amount for each primer set. Fetal levels are denoted with hatched bars while adult levels are represented by solid bars.

In order to compare the levels of H2A.Z-1 and H2A.Z-2 mRNA expression in different tissues and in HeLa cells, we performed quantitative polymerase reaction (PCR) on a panel of adult and fetal human tissue samples. Primers were designed that specifically amplify the cDNA of either H2A.Z isoform based on substantial sequence differences within the untranslated regions (UTRs). The specificity of the PCR reaction was monitored by DNA sequencing of the amplicons and by melting curve analysis. The highest levels of H2A.Z transcripts were seen in HeLa cells and in the testes (Figure 5). Figure 5 also shows that the levels of H2A.Z-1 and H2A.Z-2 transcript expression were similar in several adult tissues, including testes, ovary, prostate, peripheral blood leukocytes, small intestine, pancreas and HeLa cells. The adult brain showed approximately four times more H2A.Z-1 transcript than H2A.Z-2. A twofold increase in H2A.Z-2 transcript levels over H2A.Z-1 was seen in the adult liver and kidney. The widest range of expression levels between tissues for each variant was fivefold (liver versus testes) for H2A.Z-1 and fivefold (brain versus testes) for H2A.Z-2. In order to determine if the expression levels of the H2A.Z variants differed depending on developmental stage, we also analysed their expression in three fetal tissues for which there was an adult counterpart. When comparing the fetal H2A.Z variant transcript levels with one another, H2A.Z-1 was more abundant than H2A.Z-2 in the brain (twofold) and they had similar levels in the fetal liver and kidney (Figure 5, compare hatched bars). While the fetal kidney showed a similar H2A.Z-1 and H2A.Z-2 expression pattern compared to adult kidney (Figure 5), the patterns observed in brain and liver were different between fetus and adult suggesting possible developmental regulation of transcript levels (Figure 5).

### The promoter sequences of the H2A.Z-1 and H2A.Z-2 genes are substantially different

**Figure 6:**
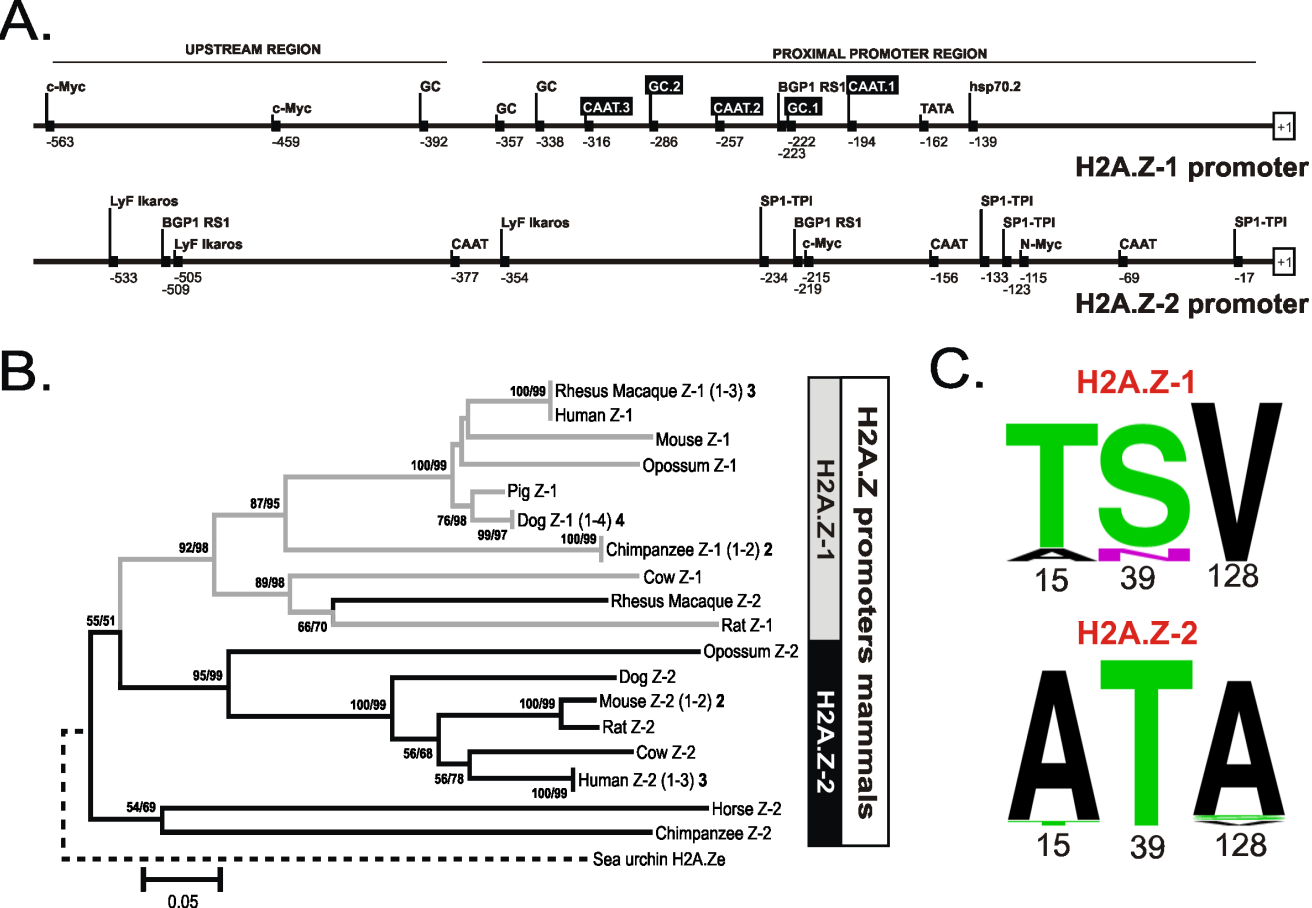
**(A)** Dissection of the putative regulatory elements in the proximal and the upstream promoter regions of H2A.Z-1 and H2A.Z-2. Elements whose relevance for H2A.Z promoter activity has been experimentally demonstrated are indicated in black boxes. The position relative to the transcription start site in the alignment, shown in Additional files 1 and 2, is indicated in each case. **(B)** Phylogenetic relationships among H2A.Z promoter regions in mammalian representatives. The numbers for internal nodes in the topology indicate confidence values for the groups defined (BS/IBT), both based on 1000 replications and only shown when a value is greater than 50%. Numbers in parentheses and in boldface near species names indicate the sequence variant copy and the number of sequences analysed, respectively (see Additional file 3). The tree was rooted with the H2A.Ze sequence from sea urchin, representing an early chordate in which H2A.Z-1 and H2A.Z-2 variants are not yet differentiated. **(C)** Logos representation of the amino acid residues at postions 15, 39 and 128 in H2A.Z-1 and H2A.Z-2. The sequences used to create the logos were the same as in **(B)**.

The evolutionary process responsible for the differentiation between H2A.Z-1 and H2A.Z-2 has been described as a refined stepwise mutation change within the codons of the three differential residues (triresidue), leading to differences in the intensity of the selective constraints acting upon the two H2A.Z isoforms in vertebrates [24]. In order to determine whether the variation in expression patterns of the H2A.Z variants could be attributed in part to differences in transcription factor binding sites in the promoters and, therefore, potential differential gene regulation, we dissected the proximal promoter regions of both H2A.Z variants in mammals (Figure 6A), where the evolutionary differentiation between H2A.Z-1 and H2A.Z-2 has reached its maximum. Recurrent search rounds for transcription regulatory elements were performed on H2A.Z promoter regions from human, rhesus monkey and mouse, leading to the identification of several putative promoter elements as well as to the localization of previously studied modules shown to be critical for H2A.Z-1 promoter activity [35]. However, comparisons between H2A.Z-1 and H2A.Z-2 promoter regions revealed completely different promoter architectures (see Additional Files 1 and 2). H2A.Z-1 shows the presence of typical elements common to other replication-independent histone variants as well as putative binding sites for other transcription factors. A perfect TATA box, three CAAT boxes and several putative GC-boxes (among which GC.1, GC.2 and GC.3 have been previously reported to form complexes with the Sp1 transcription factor) are observed in the proximal promoter region. Among these elements, one CAAT box (CAAT.2) and a GC-box (GC.2) are critical for H2A.Z promoter activity [36]. Furthermore, binding sites for c-myc are present within the upstream region of the H2A.Z-1 promoter (-459, -563) where they have been shown to specifically bind MYC and increase H2A.Z-1 transcription in response to estrogen [37].

The characterization of the H2A.Z-2 promoter emphasizes the existence of a clear differentiation between H2A.Z-1 and H2A.Z-2. The H2A.Z-2 promoter contains no TATA box and the positions of the CAAT elements and GC-boxes do not coincide with those identified in H2A.Z-1 (Figure 6A). Furthermore, it seems that H2A.Z-2 promoters show a lesser degree of conservation across the mammalian species studied, as indicated by the lack of the positional consensus in the elements identified (see Additional files 1 and 2). Searches for potential regulatory elements in the proximal promoter region resulted in the identification of three CAAT elements as well as several binding sites for transcription factors including c-Myc and N-Myc. However, the positioning of these latter binding sites at the proximal region suggests that they may function in constitutive gene expression rather than in inducible regulation in response to agents such as hormones [36,37].

The differentiation between the H2A.Z-1 and H2A.Z-2 promoters has also been analysed from a phylogenetic perspective (Figure 6B). Our results reveal the differentiation of two major evolutionary lineages based on H2A.Z promoter sequences, one encompassing the H2A.Z-1 promoter sequences and the other including H2A.Z-2 promoter sequences (Figure 6B). Such topology is in agreement with the phylogenetic inferences reconstructed on the basis of H2A.Z protein sequences and nucleotide coding regions, which also show an evolutionary differentiation between H2A.Z-1 and H2A.Z-2 variants. The presence of such differentiation at promoter regions strongly supports the presence of evolutionary constraints which act with different direction and intensity on the H2A.Z-1 and H2A.Z-2 proteins.

## Discussion

H2A.Z is the most extensively studied histone variant and it has been shown to be involved in several seemingly unrelated and divergent processes. Understanding how this protein participates in different cellular events has undoubtedly been further complicated by the use of different biological systems, since, while certain general functions of H2A.Z may be universal in all organisms, the specific details and fine-tuning in higher eukaryotes may not be present, or may be the work of other proteins, in yeast or flies. Part of this fine-tuning of H2A.Z in vertebrates could be due to the co-existence in the cell of two H2A.Z protein isoforms (H2A.Z-1/H2A.Z-2) that was first determined by mass spectrometry in total H2A.Z isolated from chicken erythrocytes [23]. An evolutionary and phylogenetic analysis of these isoforms revealed a substantial conservation at the protein level where they differ by only three amino acids (see Figure 6C), but a marked divergence at the nucleotide sequence level [24]. It also revealed that the two H2A.Z isoforms had a common origin early in deuterostome evolution that was followed by a subsequent process of differentiation [24]. Indeed, it appears that the strength of the evolutionary constraints operating at the nucleotide level are not equal between the isoforms, with H2A.Z-2 being significantly more constrained than H2A.Z-1 [24]. Cumulatively, the evolutionary evidence points toward a possible functional diversification of these two proteins.

Recently, our group has characterized the sites of acetylation within the N-terminus of H2A.Z-2 from chicken erythrocytes and from chicken erythroleukemic (MSB) cells treated with sodium butyrate to inhibit histone deacetylases [27]. Notably in the chicken, H2A.Z-2 is the variant that has traditionally been called H2A.Z or H2A.F/Z. In mouse and human H2A.Z-1 has customarily been considered H2A.Z, while H2A.Z-2 was previously known as H2A.V/F. In the current work, we determined that H2A.Z-1 is acetylated at at least three lysine residues within the N-terminus, as is H2A.Z-2. The patterns of acetylation are similar for both isoforms, where a triply-acetylated (K4, K7 and K11) form as well as doubly-acetylated (K4 + K7, K7 + K11, and K4 + K11) forms of the N-terminal peptides were identified within the sample. Singly-acetylated N-terminal peptides were also detected. However, the sites of acetylation were not able to be determined owing to the low abundances of these species. In a previous study, we identified multiple singly-acetylated H2A.Z-2 peptides, with the abundance of acetyl K4-containing species being relatively equal to the acetyl K7 species [27], and this is likely to be the case with the singly-acetylated forms of H2A.Z-1. Previously, H2A.Z-1 has been shown to be monoubiquitinated at one of several lysine residues within the C-terminus, as well as SUMOylated. However, neither modification was observed on either H2A.Z variant in chicken cells, although this could be a result of the purification method used here, or the cell type [30,38].

Our results regarding the nuclear localization of the H2A.Z isoforms indicate that they are identically distributed mainly in the euchromatin of mouse embryonic fibroblasts, as seen by other groups [6,30]. However, both H2A.Z-1 and H2A.Z-2 staining are also present within DNA-dense regions that represent the centres of chromosomes. This is most probably due to the known presence of H2A.Z in pericentric and centric chromatin [4,5]. This distribution of H2A.Z is corroborated by our biochemical analysis of chromatin fractions indicating that endogenous H2A.Z is present in greater amounts within the S1 (euchromatin) and SE (heterochromatin) fractions compared to the Pellet (insoluble chromatin). In accordance with the distribution of endogenous H2A.Z within these fractions, we see that both stably transfected H2A.Z-1-Flag and H2A.Z-2-Flag are also present in all fractions. However, interestingly, H2A.Z-2-Flag protein is present in greater amounts within the S1 fraction compared to H2A.Z-1-Flag. This suggests that H2A.Z-2 may play a greater role in the function of H2A.Z within the nuclease accessible euchromatin than does H2A.Z-1. Several systems have shown that H2A.Z functions in part to poise promoter chromatin for transcriptional activation, since it is remodelled from promoters of actively transcribing genes [10-13,39,40]. H2A.Z functioning in this manner would probably fractionate within the S1 fraction, since this chromatin would be in a more open conformation. Furthermore, the low levels of H2A.Z present within the P fraction also agree with its not playing a significant role after the initiation stage of transcription. Therefore, in HEK 293 cells H2A.Z-2 may be playing a greater role than H2A.Z-1 in poising chromatin for transcription, which agrees with our results indicating that H2A.Z-2 also shows a greater association with H3 Tri-Me K4 than H2A.Z-1 (see below).

Genome-wide distribution patterns indicate that H3 Tri-Me K4 and H2A.Z are both present at the promoters and 5' ends of genes in yeast and humans [8]. Therefore, it is not surprising that H2A.Z and H3 Tri-Me K4 are present within the same nucleosome [30,31]. The fact that H2A.Z-2 associates with H3 Tri-Me K4 to a greater extent than H2A.Z-1 is interesting because it indicates that there is a greater overlap in the regions of chromatin where H2A.Z-2 is incorporated in conjunction with the H3 Tri-Me K4 mark than H2A.Z-1. This reinforces the idea that H2A.Z-1 and H2A.Z-2 could be incorporated at different locations within the genome. The remodelling of H2A.Z into chromatin in mammals is known to occur via the action of several distinct complexes that contain the SRCAP protein, p400 or TIP 48/49 as the adenosine triphosphate-dependent remodelling subunits and evidence suggests that the deposition patterns of these complexes may differ [39,41-43]. It is tempting to speculate that one complex may prefer a specific isoform over the other, or that one complex may be responsible for incorporating H2A.Z at low levels across the genome and another for the increased levels of H2A.Z that are necessary to promote appropriate chromatin architecture at promoters [44]. Furthermore, H2A.Z nucleosomes flank nucleosome free regions whose formation is DNA-sequence dependent in several organisms including humans [8]. Whether one of the human complexes that integrates H2A.Z into chromatin contains a subunit that recognizes a specific DNA sequence is unclear but points to the potential for more than one mechanism to specify the location of H2A.Z nucleosomes [45]. Indeed, a recent *in silico *analysis suggests that both genetic and epigenetic factors can predict whether a nucleosome will contain H2A.Z [46].

The functional output of the increased association of H2A.Z-2 with H3 Tri-Me K4 is unclear, but it is highly unlikely that it results in a structural alteration of the nucleosome that would otherwise not be observed in those containing H2A.Z-1 with H3 Tri-Me K4 given the high sequence similarity of the isoforms. At least one of the effects of having H2A.Z and H3 Tri-Me K4 within the same nucleosome could be related to the recruitment or function of the RNA Pol II complex, since both the C-terminal region of H2A.Z and H3 Tri-Me K4 have been shown to interact with components of this complex [47,48]. Another possibility is that nucleosomes containing both H2A.Z and the H3 Tri-Me K4 mark protect less DNA than other nucleosomes, making the DNA more accessible for transcription [21,49]. Nevertheless, it is possible that any differences in the genomic localization and association with post translationally modified forms of other histones of the endogenous H2A.Z-1 and H2A.Z-2 could reflect spatial and temporal differences in their nuclear availability as determined by their levels of expression, or by their import into the nucleus.

Our results also reveal that H2A.Z-1 and H2A.Z-2 nucleosomes are enriched in H3 phosphorylated at S10 compared to H2A nucleosomes in asynchronous and mitotic HeLa. This association is interesting because like H2A.Z, H3 PhosS10 has been shown to be involved in processes requiring open and condensed chromatin, namely in transcription and chromosome condensation during mitosis [50]. The structural and functional consequences of H3S10 phosphorylation are not entirely clear; however, this modification does correlate with increased expression of immediate early genes after induction of MAP Kinase cascades [51,52]. Furthermore, it is not surprising that H4AcK16 is found to associate with H2A.Z-1 and H2A.Z-2 nucleosomes because it has been shown that this modification of H4 is required for incorporation of H2A.Z into subtelomeric chromatin in budding yeast [53].

Our analysis of the transcript levels of H2A.Z-1 and H2A.Z-2 indicate that there is no one dominant form, rather they are both expressed across a wide range of human tissues and that the expression levels vary depending on developmental stage. This pattern of expression probably reflects the significant sequence variation within the promoter regions of these genes and the differences in abundance of specific transcription factors within the tissues. However, it could also reflect subtle changes in the requirements of the tissues for one isoform over the other, based on any functional differences between H2A.Z-1 and H2A.Z-2. Indeed, it has recently been shown that H2A.Z-2 is specifically upregulated during macrophage differentiation and activation [54] and our analysis of the promoter of H2A.Z-2 indicates the presence of putative binding sites for the Ikaros transcription factor which is known to play a role in hematopoietic cell development [55]. Also as previously mentioned, genetic knockout studies in mice show that H2A.Z-1 is essential for development, indicating that H2A.Z-2 cannot compensate for the loss of H2A.Z-1 at least at early stages of mouse embryo differentiation [26].

The evolutionary evidence presented here suggests that it is highly possible that H2A.Z-1 and H2A.Z-2 appeared in vertebrates as a result of the entire genome duplication that took place between the divergence of the prochordates from the ancestral chordate lineage and the evolution of vertebrates, as stated by the 2R hypothesis [56]. While most of the genes arising from this duplication event were silenced, some of them were retained and their protein products either acquired a neofunctionalization (a completely new function) or a subfunctionalization in which the two new proteins acquired specialized functions [57]. This latter situation appears to be the case in going from H2A.Z-e to H2A.Z-1 and H2A.Z-2 in vertebrates.

## Conclusions

We present evidence that the two forms of H2A.Z present in mammals display similar genomic localization patterns mainly within euchromatin but that subtle differences in their association with post translationally modified forms of other histones exist. Thus, it is possible that throughout the course of vertebrate evolution, the two H2A.Z isoforms have acquired a degree of independent function that may contribute to the increased complexity and large diversity of roles for this histone variant in higher organisms. It is also possible that differences in chromatin localization patterns of the isoforms could be due to the temporal regulation of their genes, especially considering the dissimilarity in the promoter regions.

## Methods

### Immunofluorescence

Mouse embryonic fibroblasts were grown in Dulbecco's modified eagle medium (DMEM) with 10% fetal calf serum, plated on glass coverslips and allowed to attach and grow until between 40% and 80% confluent. The coding region of H2A.Z-1 was fused in frame to the 3' end of cyan fluorescent protein (CFP) into the pAmCyan1-N1 vector (Clontech) and the coding region of H2A.Z-2 was fused in frame to the 3' end of yellow fluorescent protein (YFP) into the pZsYellow1-N1 vector (Clontech, CA, USA). The cells were then co-transfected with H2A.Z-1-CFP and H2A.Z-2-YFP using effectene transfection reagent (Qiagen, CA, USA) according to the manufacturer's instructions. The cells were grown for an additional 16-20 h followed by replacement of the medium and the addition of 1 μg/ml Hoechst 33342. After 10 min, the medium was replaced with fresh medium containing no Hoechst dye. The coverslips were then mounted onto glass slides after using vacuum grease to create a small reservoir for media and imaged immediately using a Zeiss Axiovert 200 M inverted fluorescence microscope. Images were collected with a 100 × 1.4 N.A. PlanApo objective and a Photometrics CoolSnap fx CCD.

### Native H2A.Z purification

Native H2A.Z was purified from chicken MSB cells (chicken erythroleukemic cells transformed by Marek's virus) treated with 5 mM sodium butyrate for 16 h as in [58]. Isolated nuclei were extracted with HCl to solubilize the histones as described in [59]. Approximately 30-40 mg of total histones was loaded onto a 1.5 cm × 120 cm BioGel P-60 (Bio-Rad) column and eluted in 50 mM NaCl and 20 mM HCl [60]. H2A.Z eluted within a peak that also contained H2A and these fractions were selected for further purification by several rounds of reverse phase HPLC on a C18 column (Vydac, GA, USA).

### Mass spectrometry

Upon purification of H2A.Z, as described above, the H2A.Z fraction purified from sodium butyrate treated MSB cells was lyophilized and reconstituted in 100 mM ammonium bicarbonate, pH 8. An aliquot (10%) of each fraction was treated with propionic anhydride to derivatize unmodified ε-amino groups of lysine residues. Chemical derivatization with propionic anhydride converts the amino groups to their corresponding propionyl amides, and these methods have been detailed previously [61]. In brief, equal volumes of propionylation reagent (15 μL) and H2A.Z (15 μL) were reacted and derivatization was performed twice to ensure full conversion. The sample was vacuum-dried after each derivatization. The derivatized H2A.Z samples were then digested with 4 ng trypsin (Promega, WI, USA) for 8 h at 37°C. Derivatization blocks lysine residues from cleavage and, thus, trypsin cleaves C-terminal to arginine residues only. Following digestion, the samples were again reacted with propionic anhydride to derivatize the amino-termini of the trypsin-generated H2A.Z peptides. Both samples were dried a final time in a speed-vac concentrator, and were subsequently reconstituted in 0.1% acetic acid.

The resulting H2A.Z peptide mixtures were analyzed via tandem MS/MS. Each sample was pressure-loaded onto a capillary pre-column (360 μm outside diameter [(OD] × 75 μm inside diameter [ID] fused silica) packed with 4 cm of C18 reverse phase resin (5-20 μm diameter). After washing for 5 min with 0.1% acetic acid, the pre-column was connected to an analytical column (360 μm OD × 50 μm ID fused silica) packed with 8 cm of C18 (5-μm diameter) resin and equipped with an electrospray emitter tip as previously described [62]. H2A.Z peptides were eluted using nanoflow HPLC with an 1100 series high-performance liquid chromography (HPLC) pump (Agilent Technologies, CA, USA) and a 60 nL/min flow rate was achieved by splitting the flow from the HPLC. The gradient consisted of 0-60%B in 50 min and 60-100%B in 10 min (solvent A: 0.1 M acetic acid, solvent B: 70% acetonitrile, 0.1 M acetic acid). Gradient-eluted peptides were ionized using an electrospray ionization source, modified for nanospray, and were analysed using a hybrid quadrupole linear ion trap Fourier transform (LTQ-FT) mass spectrometer (Thermo Scientific, MA, USA). The LTQ-FT instrument was operated with a data-dependent method, which consisted of acquisition of a full mass spectrum using the FT as analyser followed by 10 MS/MS acquisitions of the 10 most abundant ions in the initial full spectrum. Full mass spectra were acquired for m/*z *300-2000 using a resolution of 100,000. Upon collision-activated dissociation (CAD) of precursor ions, MS/MS spectra were acquired using the ion trap as the analyser. Dynamic exclusion was enabled, by which precursor ions were selected for dissociation twice within 20 s before they were added to the exclusion list for 30 s. All CAD MS/MS spectra were interpreted via manual validation for identification of the N-terminal peptides of both H2A.Z isoforms. Accurate mass measurements acquired in the FT were utilized to generate selected ion chromatograms (SICs). A window of +/- 0.005 Da around the theoretical monoisotopic m/*z *values of the [M+2H]^+2 ^and [M+3H]^+3 ^ions was used to generate the SICs for the N-terminal peptides of H2A.Z-1 and H2A.Z-2.

### Stable transfection and chromatin fractionation

H2A, H2A.Z-1 and H2A.Z-2 sequences were amplified from HeLa cell cDNA and Flag tags were added in frame to the 3' ends by PCR. The sequences were then cloned into pcDNA3.0 vector (Invitrogen, CA, USA) and the plasmid was linearized by digesting with Sca1. HEK 293 cells were transfected using PolyFect reagent (Qiagen) and cells stably expressing the Flag-tagged protein were selected for by the addition of G418 (Gibco, CA, USA) to cell culture medium (DMEM containing 10% fetal bovine serum). After several weeks, stable colonies expressing similar levels of the proteins were selected. Nuclei were isolated and digested with Micrococal Nuclease (Worthington, NJ, USA) at 30 U/mg of DNA for 5 min at 37°C. The reaction was stopped by the addition of EDTA to a final concentration of 10 mM on ice, and the sample was centrifuged at 10,000 g for 10 min at 4°C to yield an S1 supernatant and a pellet. The pellet was resuspended and lysed in 0.25 mM EDTA pH 8 and stirred for 1 h at 4°C. Upon centrifugation as before, a supernatant SE and a final pellet P were thus obtained. Under the experimental conditions used here, S1, SE and P correspond to approximately 5%-10%, 25%-30% and 60%-65%, respectively, of the total nuclear DNA. Western Blotting was performed on the normalized protein component of these fractions using SDS-PAGE and standard procedures. Flag antibody (Sigma, NY, USA) dilution was 1:5000, H2A.Z antibody (Abcam) dilution was 1:1000, H3 Tri-Me K4 antibody (Millipore, MA, USA) dilution was 1:5000, and H4 antibody was made in-house and used at a 1:10,000 dilution.

### Preparation of mononucleosomes and immunoprecipitation

Mononucleosomes were prepared from HeLa cells transiently transfected with H2A-Flag, H2A.Z-1-Flag or H2A.Z-2-Flag within the pcDNA3.0 vector following the procedure of Sarcinella and colleagues [30]. When required, cells were treated with 100 mg/ml nocodazole for 16 h after 24 h of transfection and an aliquot was monitored by fluorescence acitvated cell sorting after treatment with propidium iodide to ensure mitotic arrest. Briefly, isolated nuclei were digested with Micrococal Nuclease (Worthington) at a concentration of 100 U/mg of DNA at 37°C for 30 min. The reaction was stopped by the addition of EGTA to a final concentration of 1 mM and the suspension was centrifuged at 600 g for 10 min. The resulting pellet was resuspended in 20 mM Tris pH 7.5, 0.2 mM EGTA, 420 mM NaCl and 1.5 mM MgCl_2 _and incubated on ice for 1 h then spun at 1000 g for 10 min. The supernatant was then brought to 150 mM NaCl by addition of 20 mM Tris pH 7.5, 1.5 mM MgCl_2_, 0.2 mM EGTA and 25% glycerol dropwise on the vortex. This suspension was centrifuged at 1000 g for 10 min and monitored on 4% native acrylamide gels to ensure complete digestion of the chromatin to mononucleosomes. For the immunoprecipitation, 10 ul of anti-Flag agarose beads (Sigma) was used to immunoprecipitate Flag-containing mononucleosomes from H2A-Flag, H2A.Z-1-Flag, H2A.Z-2-Flag or mock transfected control HeLa cells. The beads were washed eight times in 1 ml 20 mM Tris pH 7.5, 150 mM NaCl, 1.5 mM MgCl_2_, 0.2 mM EGTA, 0.2% Triton X-100, resuspended in SDS sample buffer without β-mercaptoethanol, boiled, followed by the addition of β-mercaptoethanol to the supernatant. Immunoprecipitated nucleosomes were run on 15% SDS-PAGE and transferred to polyvinylidene fluoride membrane (BioRad, CA, USA). Anti H3 AcK9K14, anti H3 Tri-MeK27, anti H3 PhosS10 and anti H4 AcK16 were all from Millipore and used at a 1:1000 dilution. Anti H2A antibody was from Abcam and used at 1:1000 dilution.

### Two-dimensional PAGE

Total histones from HeLa cell nuclei were extracted in 0.6N HCl as described in [59]. These histones were electrophoresed on a 10% polyacrylamide AUT gel [63] in several lanes, one of which was cut out and soaked in 125 mM Tris-HCl pH 6.8, 4% SDS, 20% glycerol and 1.43 M β-mercaptoethanol for 10 min at room temperature while the other was stained with Coomassie blue solution. The unstained gel strip was laid horizontally and electrophoresed in a 6% polyacrylamide stacking, 15% polyacrylamide separating SDS gel prepared according to [64].

### Quantitative PCR

Samples from three human cDNA panels derived from various human fetal and adult tissues were obtained from Clontech Laboratories Inc (CTL; CA, USA). These included normal tissue from adult (CTL MTC panel I, CTL MTC panel II) and fetal (CTL MTC fetal MTC panel) sources. Each cDNA sample represented a pool of individuals of either gender. For quantitative PCR (QPCR) analysis, cDNA was diluted 20-fold in RNase/DNAse free water. H2A.Z-1- and H2A.Z-2-specific primers were designed based on the 5' and 3' untranslated regions and amplicon sequences were confirmed by DNA sequencing. H2A.Z-1 forward primer: TTGCTTGAGCT TCAGCGGAATT, reverse primer: TTCCTTGTTATCTCAGGACTCT H2A.Z-2 forward primer: GCGGCCGAGCGGAGGCGGAG, reverse primer: TGCTTAGAGGGATGCTTTAAC. The levels of H2A.Z transcripts were analysed by SYBR Green incorporation using a Stratagene MX3005P QPCR system and MXPro software. Each 15 μl DNA amplification reaction consisted of 2 μl of diluted cDNA and 13 μl of Platinum SYBR Green qPCR SuperMix-UDG with Rox (Invitrogen) containing 2.5 pmol of each primer. Thermocycling conditions for both primer sets were: 9 min 95°C followed by 40 cycles of 15 s 95°C, 30 s 60°C and 45 s 72°C. Reactions were performed in quadruplicate and averaged cycle threshold was converted to transcript copy number by interpolation from a standard curve. The standard curve was constructed using a dilution series of purified H2A.Z-1 or H2A.Z-2 amplicon.

### Phylogenetic analysis

Nucleotide sequences corresponding to promoter regions of H2A.Z-1 and H2A.Z-2 histones were retrieved from the GenBank database through recurrent BLAST searches performed on general nucleotide collections as well on completed genomes [65]. A total of 27 nonredundant sequences belonging to mammals were compiled and properly classified as either H2A.Z-1 or H2A.Z-2 (see Additional File 3). Multiple alignments of the promoter sequences were conducted using the CLUSTAL W and the BIOEDIT programs [66,67]. Conserved regulatory elements have been simultaneously identified from the alignments of mammalian H2A.Z-1 and H2A.Z-2 sequences by using the program Transcription Regulatory Element Search (TRES, http://bioportal.bic.nus.edu.sg/tres/) to perform searches in the object-oriented transcription factors database (ooTFD) [68]. Phylogenetic relationships among H2A.Z promoter regions were reconstructed using the neighbour-joining method using uncorrected nucleotide *p*-distances using the complete deletion option. The reliability of the resulting topology was tested by both the bootstrap and the interior-branch test methods, producing the BP and CP values, respectively, for each interior node after 1000 replicates [69]. The tree was rooted with the H2A.Ze sequence from sea urchin, representing an early chordate in which H2A.Z-1 and H2A.Z-2 variants are not yet differentiated [24].

## Abbreviations

AUT: Acetic acid-urea-triton; CAD: collision-activated dissociation; CFP: cyan fluorescent protein; DMEM: Dulbecco's modified eagle medium; ID: inside diameter; OD: outside diameter; LT-FT: linear ion quadrupole trap Fourier transform; QMS: mass spectrometry; PAGE: polyacrylamide gel electrophoresis; PCR: polymerase chain reaction; QPCR: quantitative PCR; SDS: sodium dodecyl sulphate; SIC: selected ion chromatogram; UTR: untranslated region; YFP: yellow fluorescent protein.

## Authors' contributions

DD drafted the manuscript, purified native H2A.Z protein, created tagged constructs (as well as TI), performed all transfection and QPCR experiments and created the logos figure. KLR, JS and DFH performed all MS analysis. JMEL performed the evolutionary analysis. DM and MJH created microscopy images. NV and CCH provided material and aided in design of QPCR experiments.

## Supplementary Material

Additional file 1 **Nucleotide alignment of H2A.Z-1 and H2A.Z-2 proximal promoter sequences from representative mammals (human, rhesus monkey and mouse)**. Regulatory sequences in H2A.Z-1 are indicated by solid boxes in yellow (TATA box), green (GC box) and blue (CAAT box). Regulatory elements in H2A.Z-2 are underlined in red (SP1 TP1 binding sites) and purple (c-Myc binding sites). Numbering above the alignment represents the number of nucleotides from the origin of transcription. Click here for file

Additional file 2 **Nucleotide alignment of H2A.Z-1 and H2A.Z-2 upstream promoter region sequences from representative mammals (human, rhesus monkey and mouse)**. Regulatory elements in H2A.Z-1 are indicated by solid boxes in red (GC boxes), blue (CAAT boxes), purple (c-Myc binding sites), and by an open box (BGP1 RS1 binding site). Regulatory elements in H2A.Z-2 are underlined in red (SP1 TR1 binding sites), purple (c-Myc binding sites), blue (CAAT boxes), green (LyF Ikaros binding site), brown (N-Myc binding sites) and by open boxes (BGP1 RS1 binding sites). Numbering above the alignment represents the number of nucleotides from the origin of transcription. Click here for file

Additional file 3 **GenBank Accession numbers for the histone variants H2A.Z-1 and H2A.Z-2 used in the present work**. The ANNOTATION field denotes: gene sequences newly isolated from draft genomes (*in silico*), gene sequences predicted as either H2A.Z-1 or H2A.Z-2 from databases and draft/complete genomes data (Pred), sequences defined either as H2A.Z-1 or H2A.Z-2 by the present analyses (a), sequences defined as H2A by the present analyses (b) and sequences whose annotation either as H2A.Z-1 or H2A.Z-2 has been corrected by the present work (c). Click here for file
