# Supplementary figures and images for: Characterization of the histone H2A.Z-1 and H2A.Z-2 isoforms in vertebrates
Source: BMC Biol. 2009 Dec 14;7:86. doi: 10.1186/1741-7007-7-86 (PMC2805615; doi:10.1186/1741-7007-7-86)

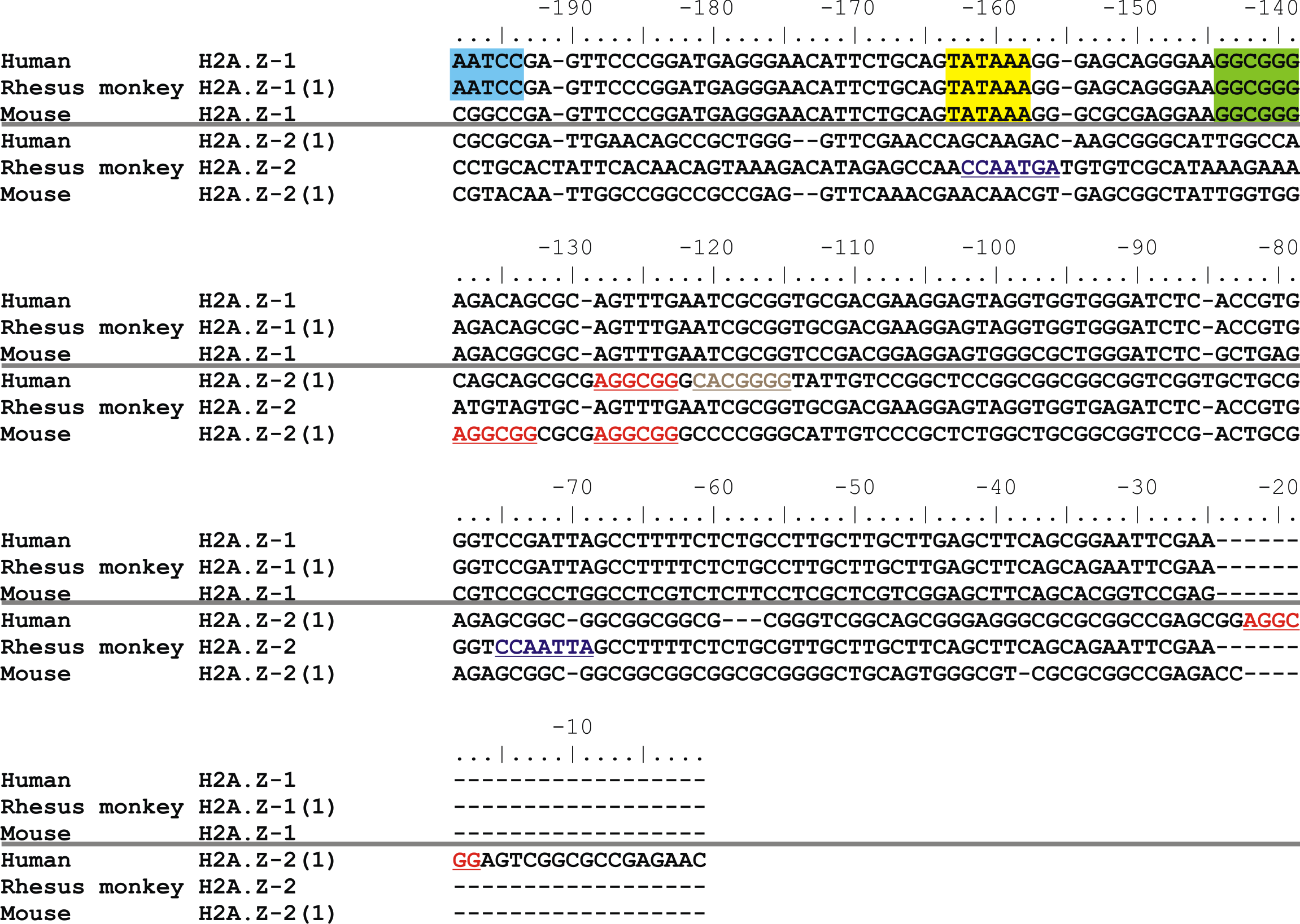

Supplement: Additional file 1 — Nucleotide alignment of H2A.Z-1 and H2A.Z-2 proximal promoter sequences from representative mammals (human, rhesus monkey and mouse). Regulatory sequences in H2A.Z-1 are indicated by solid boxes in yellow (TATA box), green (GC box) and blue (CAAT box). Regulatory elements in H2A.Z-2 are underlined in red (SP1 TP1 binding sites) and purple (c-Myc binding sites). Numbering above the alignment represents the number of nucleotides from the origin of transcription. [file 1741-7007-7-86-S1.PNG]

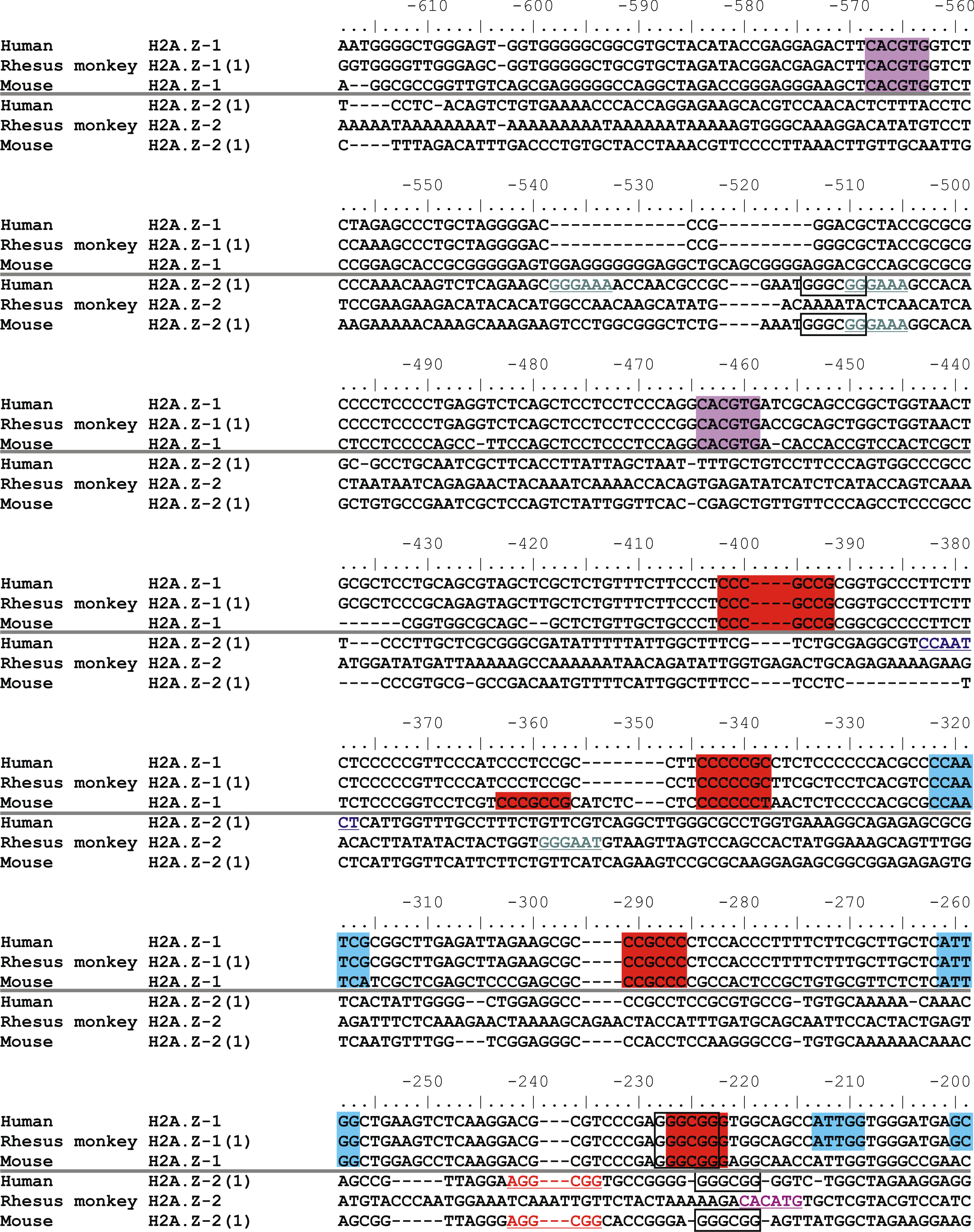

Supplement: Additional file 2 — Nucleotide alignment of H2A.Z-1 and H2A.Z-2 upstream promoter region sequences from representative mammals (human, rhesus monkey and mouse). Regulatory elements in H2A.Z-1 are indicated by solid boxes in red (GC boxes), blue (CAAT boxes), purple (c-Myc binding sites), and by an open box (BGP1 RS1 binding site). Regulatory elements in H2A.Z-2 are underlined in red (SP1 TR1 binding sites), purple (c-Myc binding sites), blue (CAAT boxes), green (LyF Ikaros binding site), brown (N-Myc binding sites) and by open boxes (BGP1 RS1 binding sites). Numbering above the alignment represents the number of nucleotides from the origin of transcription. [file 1741-7007-7-86-S2.PNG]
