# Supplementary material for: Characterization of the histone H2A.Z-1 and H2A.Z-2 isoforms in vertebrates
Source: BMC Biol. 2009 Dec 14;7:86. doi: 10.1186/1741-7007-7-86 (PMC2805615; doi:10.1186/1741-7007-7-86)
Supplement: Additional file 3 — GenBank Accession numbers for the histone variants H2A.Z-1 and H2A.Z-2 used in the present work. The ANNOTATION field denotes: gene sequences newly isolated from draft genomes (in silico), gene sequences predicted as either H2A.Z-1 or H2A.Z-2 from databases and draft/complete genomes data (Pred), sequences defined either as H2A.Z-1 or H2A.Z-2 by the present analyses (a), sequences defined as H2A by the present analyses (b) and sequences whose annotation either as H2A.Z-1 or H2A.Z-2 has been corrected by the present work (c). [file 1741-7007-7-86-S3.DOC]

| **SPECIES** | **GENE** | **CHROMOSOME** | **ACCESSION NUMBER** | **ANNOTATION** |
| --- | --- | --- | --- | --- |
| Bos taurus (Cattle) | H2A.Z-1 | 4 | NM_174809 |  |
|  | H2A.Z-2 | 6 | NM_001038197 |  |
| Canis familiaris (Dog) | H2A.Z-1 (1) | 2 | XM_535390 | Pred |
|  | H2A.Z-1 (2) | 32 | XM_535671 | Pred (a) |
|  | H2A.Z-1 (3) | 32 | XM_857355 | Pred (a) |
|  | H2A.Z-1 (4) | 32 | XM_857381 | Pred (a) |
|  | H2A.Z-2 | 16 | XM_532724 | Pred |
| Equus caballus (Horse) | H2A.Z-2 | 4 | XM_001495899 | Pred (a,b) |
| Homo sapiens (Human) | H2A.Z-1 | 4 | NM_002106 |  |
|  | H2A.Z-2 (1) | 7 | NM_012412 |  |
|  | H2A.Z-2 (2) | 7 | NM_138635 |  |
|  | H2A.Z-2 (3) | 7 | NM_201436 |  |
| Macaca mulatta (Rhesus Monkey) | H2A.Z-1 (1) | 5 | XM_001108067 | Pred |
|  | H2A.Z-1 (2) | 5 | XM_001108128 | Pred |
|  | H2A.Z-1 (3) | 9 | XP_001097247 | Pred (c) |
|  | H2A.Z-2 | 9 | NC_007866 | In silico (a,b) |
| Monodelphis domestica | H2A.Z-1 | 5 | XM_001364009 | Pred (a,b) |
|  | H2A.Z-2 | 1 | XM_001379779 | Pred (a,b) |
| Mus musculus (Mouse) | H2A.Z-1 | 3 | NM_016750 |  |
|  | H2A.Z-2 (1) | 11 | XM_907680 | Pred |
|  | H2A.Z-2 (2) | 11 | XM_00147068 | Pred |
| Pan troglodytes (Chimpanzee) | H2A.Z-1 (1) | 8 | XM_001163743 | Pred (a,b) |
|  | H2A.Z-1 (2) | 8 | XM_519801 | Pred (a,b) |
|  | H2A.Z-2 | 15 | NW_001225258 | In silico (a,b) |
| Rattus norvegicus (Rat) | H2A.Z-1 | 2 | NM_022674 |  |
|  | H2A.Z-2 | 14 | NM_001106019 |  |
| Sus scrofa (Pig) | H2A.Z-1 | 8 | NM001123122 |  |
